# Is Social Contact With the Resident Population a Prerequisite of Well-Being and Place Attachment? The Case of Refugees in Rural Regions of Germany

**DOI:** 10.3389/fsoc.2020.578495

**Published:** 2020-12-18

**Authors:** Birgit Glorius, Stefan Kordel, Tobias Weidinger, Miriam Bürer, Hanne Schneider, David Spenger

**Affiliations:** ^1^Chair of Human Geography With Focus on European Migration Research, Institute for European Studies and History, Chemnitz University of Technology, Chemnitz, Germany; ^2^Department Geography and Geoscience, Institute for Geography, University of Erlangen-Nuremberg, Erlangen, Germany

**Keywords:** integration, social contact, well-being, place attachment, neighborhood, refugees, rural regions

## Abstract

Due to dispersal policies applied in many European countries, such as Germany, rural regions are important arrival regions for asylum seekers and refugees. For German policy makers, who have faced a large number of immigrants since 2015, it is crucial that asylum seekers and refugees stay in those rural regions and benefit the development of those areas. This paper addresses the quality and quantity of social contact between refugees and resident populations as a prerequisite for integration and long-term migration-development effects from a social geographical perspective. Drawing from survey data and qualitative interviews, we examine expectations, perceptions and experiences of everyday encounters and social relationships in neighborhoods in small rural towns and villages from the perspective of both local residents and refugees. Our results support arguments from research literature for faster social inclusion in rural areas due to greater nearness, but also obstacles toward the integration of foreigners due to a higher homogeneity of rural neighborhoods and only few experiences of positive everyday contact with foreigners among rural residents. The interviewed refugees display a high level of reflexivity regarding their new neighborhood and how they might be seen by rural residents. Their experiences encompass various forms of social relationships, while social bridges are crucial, ranging from serendipitous encounters and functional interactions to connections based on mutual interest around family issues or cultural aspects. Openness and tolerance from at least some parts of the local population can help immigrants to feel at home, and support staying aspirations, while simultaneously evoking wider social change. A peculiarity of rural areas is the intersectionality with further challenges related to structural changes, encompassing, for instance, socio-demographic and economic restructuring. However, social interactions and opportunities for encounters are only one factor in the development of long-term settlement. More in-depth research is needed to consider the interrelations of both structural contexts and complex and changing needs for personal development in the future, also from an intergenerational perspective.

## Introduction

In 2015 and 2016 there was an unprecedented rise in the number of asylum seekers arriving in Germany, which presented a challenge for the reception system and required strong efforts for long-term integration. While rural regions are usually not in the primary focus of immigrants, they host a considerable share of the world's refugee population. This is mostly due to state distribution mechanisms meant to share the burden of reception and integration, such as the quota system “Königsteiner Schlüssel” applied in Germany. Thus, of the 1.8 million people who sought asylum in Germany between 2013 and 2018 (Bundesministerium des Innern, für Bau und Heimat, 2020), roughly 43% today live in rural regions (own calculations, based on the typology provided by Küpper, 2016). In the public debate accompanying the allocation process, it was argued that rural regions could benefit from the surplus population, as those mostly young and partly well-educated migrants could help to overcome the effects of demographic aging and labor shortages, notably in those rural arrival regions that have faced a strong demographic decline for decades (Braun and Simons, 2015; Empirica, 2016; Franke and Magel, 2016; Weidinger, 2020). Thus, immigration was framed as a possible driver of local development (Kordel and Weidinger, 2020; Weidinger, 2020).

Exploring this argumentation from the perspective of integration theory, it is obvious that an integration outcome as envisaged above depends on a positive integration trajectory, which is not only dependent on (infra)structural aspects, such as absorptive capacities in the housing and labor market, but also on the social embeddedness of newcomers in a specific socio-spatial environment. The notion of well-being, or more precisely “social well-being,” is helpful for conceptualizing this. While well-being is usually described as a complex psychological concept, social aspects, notably the dimension of social acceptance and social integration, are seen as crucial (Carruthers and Hood, 2004; Teghe and Rendell, 2005). Research on well-being has found that the place in which one lives contributes to the level of well-being in manifold ways, be it the beauty of the nature, good neighborhood relations or the feeling that one can contribute positively to the local society (Zumbo and Michalos, 2000; Coulthard et al., 2002; Shields and Wooden, 2003). On the community level, social well-being is connected to social sustainability and resilience: social well-being of migrants in a community enlarges collective social capital, and can thus initiate, steer or intensify community development (Putnam, 1993, 2000; Teghe and Rendell, 2005). Well-being in this context must be understood as an individual psychological condition, yet embedded in time and space (see, e.g., Diener, 2009; Aikawa and Kleyman, 2019).

In this paper, we argue that positive social contact with the resident population is a prerequisite for immigrants' well-being. The socio-spatial characteristics of rural arrival regions, with a high level of interpersonal contacts and social nearness, might provide a supportive context for establishing positive social contacts and interactions between immigrants and resident population, which can gradually lead to reciprocal influence and social change (Sam, 2006, p. 15). Crucial aspects are the quantity and quality of everyday interactions between the resident population and immigrants, the openness of the resident population toward foreigners, and the willingness of both—resident population and immigrants—to integrate (Berry, 1991; Sam, 2006). This paper departs from those framing thoughts and explores mutual expectations, perceptions and experiences of social contact between residents and refugees in rural regions of Germany. The empirical part follows a multi-perspective approach and draws on empirical data from a citizen survey among 908 rural inhabitants, and from 139 qualitative interviews with refugees, which were conducted in eight rural districts in the course of a collaborative research project between 2018 and 2020 (see section Results).^1^

Our paper is structured in five sections: following this introduction (section Introduction), a conceptual chapter discusses major theoretical approaches regarding the role of social contact in integration processes and the establishment of place-based belonging as a precondition for evoking social change (section Conceptual Considerations). We then briefly explain the main steps of our empirical research (section Materials and Methods). Section Results presents our findings, regarding the expectations, perceptions and experiences of social contacts among the resident population and refugees. In the final section Discussion and Conclusion, we discuss our findings and draw preliminary conclusions for the wider field under study.

## Conceptual Considerations

Putting the individual's strive to participating in the economic, political and social life of the host society and associated societal frameworks at the core of the debate about integration, Ager and Strang (2008) developed a mid-level theory for analyzing integration, both from the perspective of immigrants, in this case refugees, and the local or resident population. Ten interdependent realms, facilitators, and key components, which are presented hierarchically, represent the core of the theory. Social connection plays an important role in, among other things, accessing employment, housing, education and healthcare, and thus drives “the process of integration at a local level” (Ager and Strang, 2008, p. 177). Thereby, a distinction can be drawn between social bonds (encompassing relations to family members, ethnic, national or religious communities) and social bridges (i.e., those with the resident population and social links, or those with actors associated to governmental structures (Putnam, 1993, 2000; Ager and Strang, 2008).

Social bonds and bridges offer practical support to refugees, e.g., with regard to access to health and welfare services, interpreting, financial and emotional support, and reducing feelings of isolation and depression (Zetter and Pear, 2000; Sales, 2002), while bonds in particular enable immigrants to share cultural and social practices and “maintain familiar patterns of relationships” (Ager and Strang, 2008, p. 178). Whether social relationships become useful for the individual and subsequently support the integration process or increase their well-being depends on the degree to which they are able to overcome social distances (Granovetter, 1973). Following Granovetter (1973), connections that go beyond a bounded social network and function most effectively when they bridge social distance are termed weak ties (Granovetter, 1983, p. 208).

Especially if migrant communities are absent or are very small in number, as is often the case in rural areas, immigrants are very much reliant on establishing contacts with the resident population to receive assistance and support upon arrival (De Lima et al., 2012). For rural areas, empirical studies on social connections highlighted the greater social proximity, which facilitates orientation and enables a high number of direct contacts (Micksch and Schwier, 2000; Schader, 2011; Gruber, 2013). There is a higher potential for community and social security, and a high degree of self-organization in associations and of volunteering, which present opportunities for social participation of newcomers and facilitate integration processes (Nadler et al., 2010; Arora-Jonsson, 2017; Priemer et al., 2017; Tesch-Römer et al., 2017; Wagner, 2019). On the other hand, however, rural societies are often associated with limited diversity, an implicit understanding of homogeneity and a rather high degree of social control, which could result in strong pressure to assimilate (Rösch et al., 2020). In addition, there is a higher sensitivity toward foreignness and difference, which is more often perceived as problematic (Gruber, 2013; Arora-Jonsson, 2017). Thus, findings point to specific assets, but also possible detriments of rural regions regarding immigrant integration, of which social contact is only one, albeit an important, building block.

### Social Contact and the Role of the Resident Population

While integration research usually focuses on the aspirations, experiences and behaviors of immigrants, it is obvious that integration efforts are strongly determined by the behaviors and intentions of the resident population too. However, their role is rarely systematically researched (De Lima et al., 2012; Phillimore, 2020). Regarding the empirical focus of this paper, acculturation psychology provides a helpful concept, as it takes both “parties” into consideration. We adopt the definition of social psychology which defines acculturation as “those phenomena which result when groups of individuals having different cultures come into continuous first-hand contact, with subsequent changes in the original culture patterns of either or both groups” (Redfield et al., 1936, p. 149). The details of acculturation are further spelled out by the contact hypothesis: While contact is seen as essential for the initiation of adaptation or change processes, it has to be first-hand, continuous, and must take place in a specific spatio-temporal setting (Allport, 1954), i.e., in regular everyday situations, such as the neighborhood, workplace or school.

Comparative empirical research on attitudes of rural residents toward immigrants reveals a reluctant or even hostile attitude, which might be due to less contact and individual experiences of living with immigrants (e.g., Czaika and Di Lillo, 2018, for European comparison; Zahl-Thanem and Haugen, 2018, for the case of Norway). A study on the social integration of Polish labor migrants on a Norwegian island stressed the level of social exposure, i.e., the likelihood of contact and the degree of intra-community contact of immigrants (as social bonds) and between migrants and resident population (as social bridges), as an element of social integration (Stachowski, 2020). Another study on immigrant integration in rural Sweden suggests that the level of “strangeness” in terms of skin color and ethnicity influences the ways residents approach newcomers, mixing othering processes on the basis of (non)whiteness with categories, such as race and class (Arora-Jonsson, 2017). The salience of “rural racism” is also addressed in the edited volume of Chakraborti and Garland (2004), while the research of Roos (2016) suggests that inter-group contacts in a neighborhood are crucial for reducing xenophobia.

Further steps in the acculturation process, following contact theory, are reciprocal influence and change. It is assumed that continuous personal contact causes mutual influence that can bring about changes in attitudes, behaviors, and also institutional change. Those change processes are conceptualized as dynamic and long-lasting, and they are not reduced to social or cultural change (Berry, 1991). However, we need to point out power asymmetries between resident and immigrant populations, which obviously influence the level and direction of change (Sam, 2006, p. 15). We find empirical evidence in the aforementioned study on immigrant integration in rural Sweden, which revealed that immigrants rarely engaged in local associations. They also hesitated to establish ethnic associations, as they feared being perceived as different and culturally incapable (Arora-Jonsson, 2017). An ecological development project initiated by an ethnic association was, while initially publicly valued, sabotaged by locals, pointing to the hegemonic attitude of the locals that assigns immigrants their place in the social fabric of the community (Arora-Jonsson, 2017).

### The Role of the “Social” Regarding Refugees' Well-Being and Place Attachment

Social networks are considered crucial for supporting refugees' psychosocial well-being and resilience (Evans, 2005) as well as for maintaining their psychological and emotional health (e.g., Kia-Keating and Ellis, 2007; Chase et al., 2008). To be in a state of well-being, Lynnebacke (2020) identified the development of emotional bonds and a feeling of place attachment as prerequisites. In conceptual terms, the notion of embeddedness describes social relationships that encourage a sense of belonging or rootedness to a local environment (Korinek et al., 2005). With the term place attachment, Lewicka (2010) introduced a deliberate affective dimension to the discussion. Place attachment is defined as “emotional ties that people develop with their places of residence” (Lewicka, 2010, p. 35). Again, in this context, social connections are crucial. Besides exposure and familiarity toward a place, more convincing explanatory factors were presented by Ehrkamp (2005) and Richter (2011). First, social connections become stronger over time, allowing individuals to ascribe social meanings to places. Second, individuals associate places with biographical events. Finally, affective embodied experiences may play a role (Lewicka, 2014; Lynnebacke, 2020).

A case study conducted by Boese and Philips (2017) in rural Australia highlighted the positive role of social contacts with rural residents and the participation in cultural practices, while spaces for interaction and shared experiences were found to be an important prerequisite for establishing attachments to places of residence (cf. Wernesjö, 2015; Boese and Philips, 2017, p. 63; Radford, 2017). Lacking spaces for interaction or not using available spaces—e.g., due to poor language proficiency, refugee parents' fear of discrimination or restrictive behavior toward their children, or negative attitudes from local residents—may undermine the individual's sense of attachment to place (cf. Hummon, 1992; Low and Altman, 1992; Schech and Rainbird, 2013). In addition, Spicer (2008) reminds us that experiences of neighborhood places and resulting effects of exclusion and inclusion are always age-specific and life-course related. In a case study carried out by Gilhooly and Lee (2017), participants in rural Georgia, USA, compared the opportunities to connect with neighbors with their former places of residence in cities, and highlighted the advantages of the countryside; these rural areas were also appreciated for raising children in a protected environment far from racial tensions and bad influences in urban areas (see also Huisman, 2011). The friendliness of the population experienced in everyday encounters, i.e., being recognized and greeted by others, positively contributes to feeling more secure (Ager and Strang, 2008).

In this section we presented key concepts related to the integration trajectories of immigrants, highlighting the development of social contact with the resident population as a “social bridge,” notably in rural and less diverse settings where migrant communities are scarce. Taking the contact hypothesis as a starting point, we identified social proximity as a peculiarity in rural areas, which can result in supportive structures, but also in social control and pressure to assimilate with local behavior and customs. The latter may stem from the observation that how and with whom people interact in rural areas is strongly shaped by the resident population and is thus often a hegemonic experience. Moreover, social proximity increases ascriptions of “otherness” and can reinforce hostile attitudes. Whilst refugees' development of emotional ties to the place of arrival is a prerequisite for sustainable local integration trajectories, following Berry (2006) model of acculturation outcomes, we address social connections in a neighborhood as an important component since spaces of interactions can create shared experiences.

## Materials and Methods

Data for this paper stem from our ongoing collaborative research project “Future for refugees in rural regions of Germany,” which aims to provide in-depth results on rural integration conditions. The project applies a mixed-methods approach, collecting structural data on major integration dimensions, such as housing, labor market, mobility, education, and health, implementing expert interviews on local integration governance, and addressing both the refugees and the residents of rural municipalities as respondents with specific perspectives on the topic. The fieldwork for this project took place in 32 rural municipalities (40 for the citizen survey), spread over eight rural districts in the Federal States of Bavaria, Hesse, Lower Saxony and Saxony. Following a typology of rurality derived by the Thuenen Institute (Küpper, 2016), all districts are classified as “very rural.”^2^ For the 32 municipalities within the districts, we only chose municipalities with <20,000 inhabitants, which were also sites of refugee reception at the time of inquiry.

The presented paper uses two datasets from this ongoing project: first, a citizen survey among 908 residents in the case study sites and, second, a series of 139 qualitative interviews with foreigners mostly holding a recognized protection status^3^ who reside at those sites. Such a mixed methods approach was applied since two target groups were addressed: for an informative sample size for the survey, a postal process was most promising and affordable, whilst for the perspective of refugees, language barriers and challenges to access them made face-to-face encounters the most valuable method.

### Representative Survey

The original sample size was 4,000−100 people in each of the 40 chosen municipalities, of which 32 were hosting refugees. The survey was implemented as a written survey, giving respondents the opportunity to either fill in a paper questionnaire or do an online version of the survey. The questionnaire had five sections, starting with questions on the rural living environment and neighborhood relations (with “neighborhood” defined as “your ultimate living environment”) (1) and the current economic situation and life satisfaction (2), and then moving the focus gradually toward aspects of ethnic and cultural relations with reference to several scales of observation (3), attitudes to asylum and integration (4), and concluding with questions on the socio-demographic profiles of the respondents' households (5). A pre-test was carried out to make sure that the wording of the questionnaire was understood by the survey respondents. The postal addresses were selected as a representative sample in each of the case study sites' populations registers. Due to non-response and some cases of address failure, we were able to compile 908 valid responses, which amounted to a response rate of 23%. This is satisfactory since the response rate of postal surveys strongly depends on the amount of contact, e.g., follow-up letters, personal contact and reminder letters (Menold, 2016). Due to data security reasons and limited resources, the participants received only one letter with the questionnaire and one follow-up postcard. As responses are distributed homogeneously across the survey regions and correspond to the total sample population in central socio-demographic indices, we assume that responses are representative. Nevertheless, a bias in the sample due to heterogeneous interests in (not) participating in the study cannot be ruled out. Data were coded, computed, and analyzed with the Statistical Package for the Social Sciences (SPSS). First, descriptive analyses were carried out, which will be followed by multivariate analyses later on in the project.

### Interviews With Refugees

Narrative interviews were conducted among refugees who had resided at least 6 months in Germany, and lived in one of the selected 32 municipalities when the sampling took place. Most of them had some kind of protection status at the time of the interview (see text footnote 3). Preparation for the interviews entailed icebreaker meetings to build up personal relationships between the interviewer, the participant and, where needed, the interpreter. The interviews themselves aimed at unraveling past experiences of other places since arrival in Germany, and providing an in-depth reflection about the current rural site of living and future aspirations. Following a participatory research approach, the narrative interview included two visual tools: (im)mobility biography (Kieslinger et al., 2020) and mobility mapping (Weidinger et al., 2019). With the former, participants were invited to draw their previous places of residence, either group accommodation or self-rented flats, and to reflect on them. This paved the way for an interpretation of relational negotiations of “good” places and neighborhoods. With mobility mapping, participants identified individually important places in their everyday lives and evaluated their accessibility.

In total, 139 interviews with 192 people were conducted, lasting between 60 and 235 min. Participants mostly originated from Syria (*n* = 110), Afghanistan (*n* = 22), Iraq (*n* = 19), and Eritrea (*n* = 12), and they were 34.3 years old on average. The sample included both individuals that lived alone as well as families with and without children. The perspective of women, who often arrived in the course of family reunification, was especially taken into consideration, being reflected in their 42.2% share of participants. However, for this paper, no gender-specific analysis was conducted, since the focus was on negotiations concerning overall family-related constellations. For the analysis, an emphasis was put on connections between the spoken word and the graphical elicitation. Thus, interviews were transcribed verbatim and subsequently coded, using a deductive-inductive approach with both descriptive and analytical codes. Timelines and maps were edited graphically and rendered anonymous. The analysis followed the product-oriented mode of analysis for narrative mapping suggested by Lutz et al. (2003) as well as the thematic (and visual) analysis as part of narrative analysis suggested by Kohler Riessman (2005, 2008). Quotes presented below were translated into English by the authors.

## Results

In this section, we will explore expectations for and perceptions of a positive neighborhood, i.e., the question of neighborhood quality, neighborly contact and concrete experiences. We will jointly discuss data from our citizen survey among rural residents and data from interviews with refugees on their perception of coexistence in the rural neighborhoods. Focusing on the specific rural conditions, it will be illustrated how they influence personal expectations, perceptions and experiences of neighborhood relations. As a general differentiating aspect, we have to point out that the respondents of our citizens' survey have very little direct contact with foreigners at all, while the interviewed refugees can certainly all reflect on experiences as newcomers in a rural neighborhood. This general difference leads to the situation that refugees present an informed reflection on their experiences in the neighborhood, while the survey respondents mainly stay at the level of expectations and perceptions, both of which are seemingly shaped by specific stereotypes regarding foreigners in the neighborhood.

### Expectations and Perceptions of Neighborhood Relationships: Perspectives of Resident Population and Refugees

From the side of the rural resident population, there is a high satisfaction rate regarding the neighborhood quality: a large majority of respondents either fully or mostly agreed about feeling happy in their neighborhood (90.2%) and stated that there is a nice, friendly atmosphere (85.5%). Most neighbors are known personally (84.6% fully/mostly agree), and are characterized as helpful (85.1%) (Table 1). Concerning openness toward newcomers, most respondents have the impression that integration into the neighborhood would not be difficult. Only 13.1% agree with the statement that newcomers would not have it easy in the neighborhood, and 21.4% partly agree (Table 1). However, given that most respondents have already lived in their neighborhood for quite a long time, this impression might be biased due to their own lack of experiences with moving into a new neighborhood.

**Table 1:** Conditions of the rural neighborhood (in % of responses).

**Item**	**Not at all.**	**Somewhat disagree**	**Partly yes, partly no.**	**Somewhat agree**	**Fully applies.**
There are many elderly people living in my neighborhood.	1.4	11.3	40.9	32.5	13.9
There are many families with children in my neighborhood.	10.2	33.2	34.9	13.0	8.7
There are many residents with a migration background in my neighborhood.	49.3	34.2	12.0	2.7	1.8
I feel happy in my neighborhood.	0.7	1.2	7.9	38.9	51.3
The atmosphere in my neighborhood is nice and friendly.	0.8	1.8	11.9	43.7	41.8
I mostly know my neighbors.	0.9	3.9	10.6	24.7	59.9
In general, most neighbors are helpful.	1.1	2.7	11.1	42.1	43.0
Newcomers don't have it easy in the neighborhood.	20.3	45.2	21.4	10.0	3.1

*Source: own survey, N = 803–890*.

**Figure 1:**
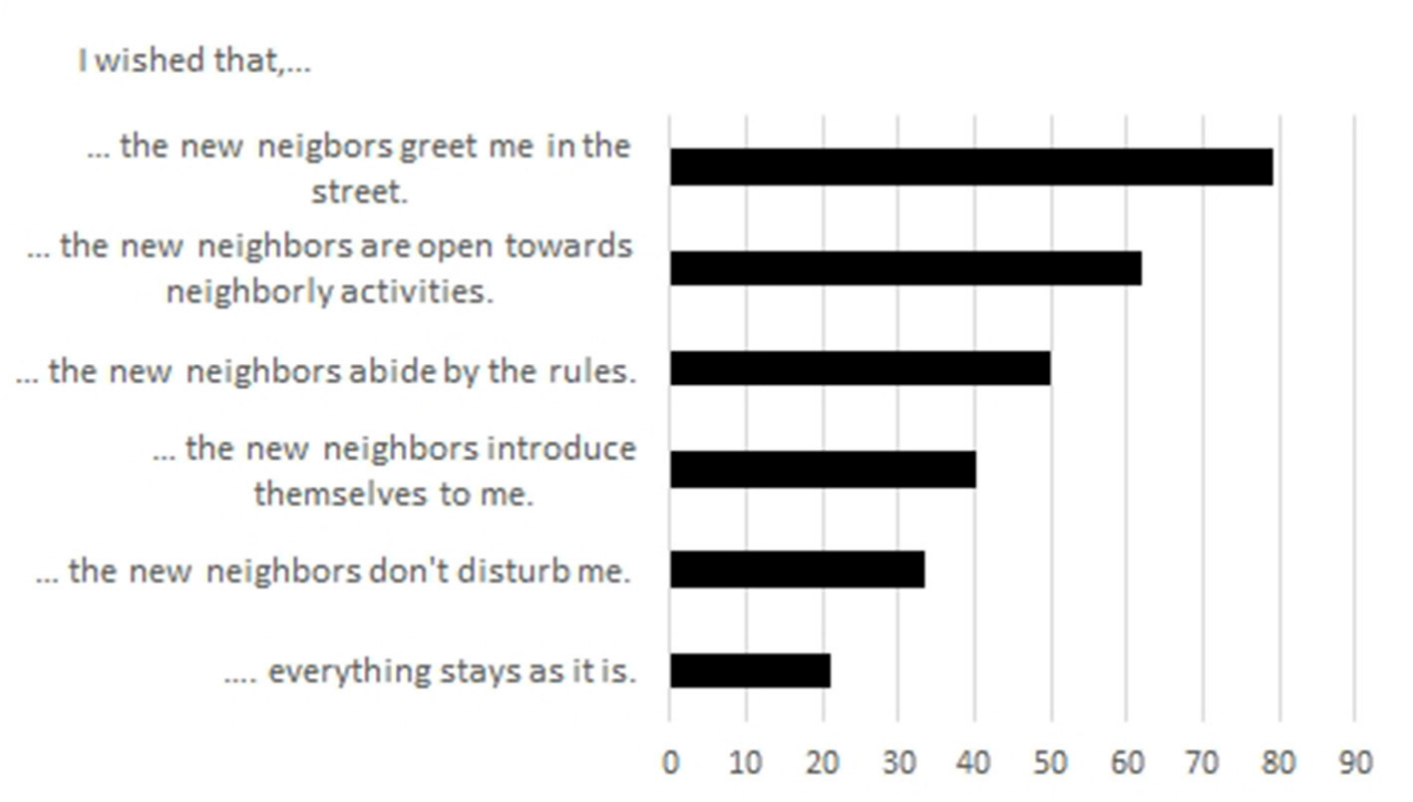
Expectations regarding newcomers in the neighborhood (in % of respondents); Source: own survey, *N* = 904.

What does the resident population expect from newcomers in their rural neighborhood? The majority (79.4%) hope that newcomers would greet them in the street. Furthermore, new neighbors should be open for neighborly activities (62.1%) and abide by the rules (50.0%). Two fifths of our respondents want newcomers to introduce themselves to them; one third hope to not be disturbed by new neighbors, and one fifth expect that nothing would change if newcomers moved into the neighborhood (Figure 1). Those answers give a good indication of the social environment in rural neighborhoods: while inhabitants' self-assessments suggest a friendly and helpful overall atmosphere, personal contacts are of minor intensity, but generally possible. While in daily life, people generally stay at a distance, they are ready to engage more intensively should the situation make it necessary. The integration of newcomers into the social fabric of the neighborhood is not perceived as problematic, as long as newcomers understand and abide by the (unwritten) rules of the society.

**Figure 2:**
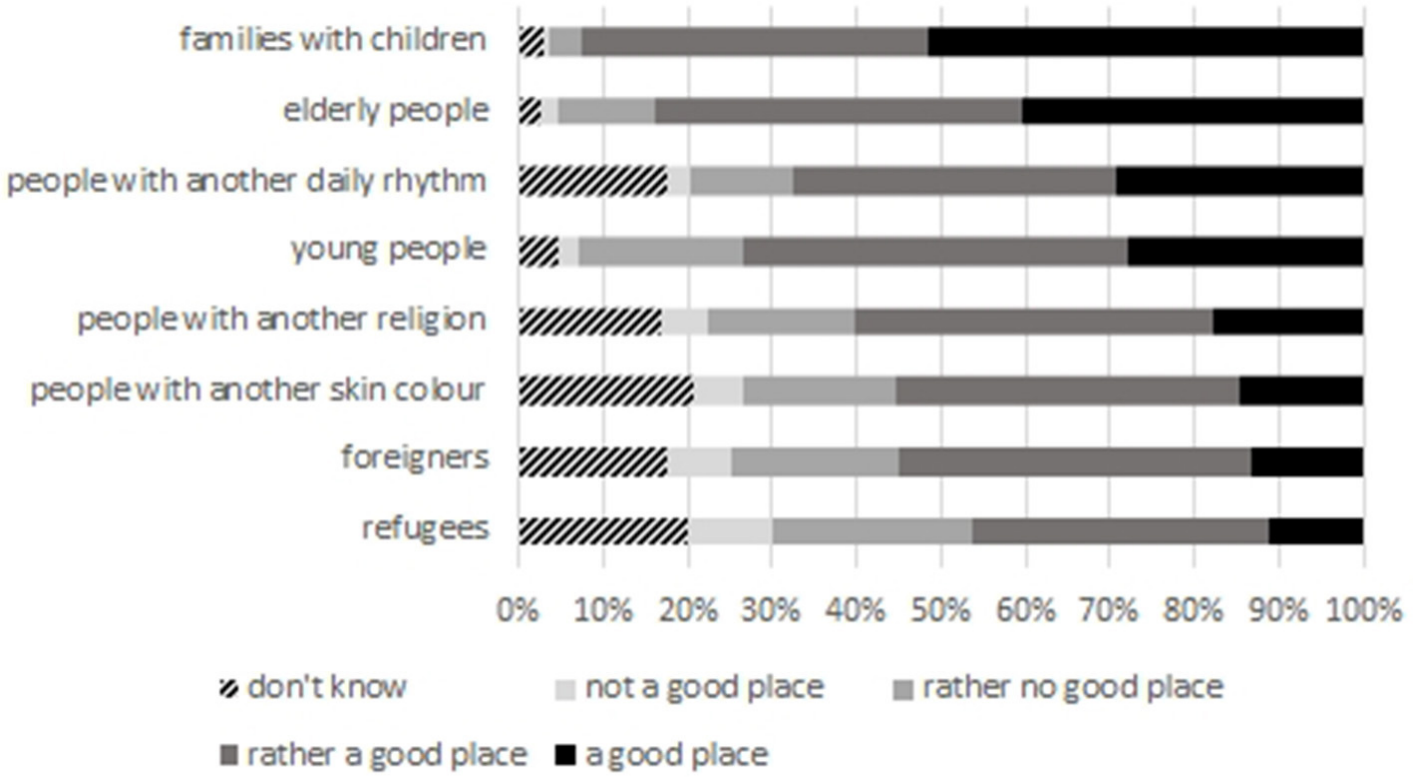
“Is your town either a good or not a good place to live for …” (in % of respondents); Source: own survey, *N* = 857–874.

However, we cannot validate if this rather open attitude would equally apply to any social group. In order to gain more insight into possible prejudices, we asked our respondents for an assessment about the suitability of their municipality regarding social groups that display a varying degree of diversity in comparison to the population majority. As the answers show (Figure 2), for groups who are actually present in the neighborhood (elderly people, families, young people), the municipality is perceived a suitable place. For groups that significantly differ from the respondents' own characteristics, the assessment is less positive, and there is a higher rate of people who cannot decide (“don't know”). This is specifically clear for “foreigners,” “people with another skin color,” and “refugees,” where around half of the respondents either make a negative assessment or cannot decide at all. The reason for this assessment might be their own feelings of hostility, but it could also be unfamiliarity with diversity in everyday life, as suggested by other empirical evidence on rural places of reception (e.g., Gruber, 2013; Arora-Jonsson, 2017).

**Figure 3:**
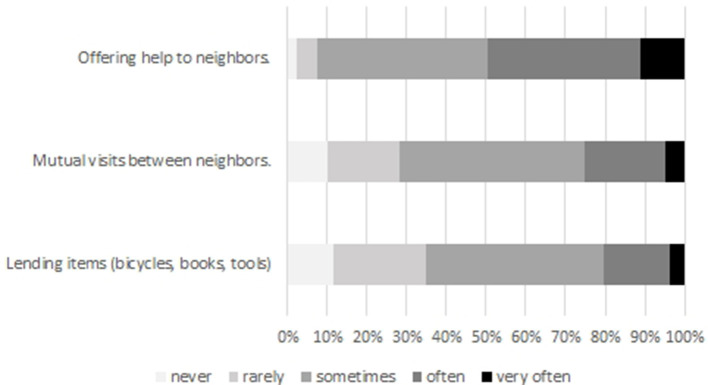
Frequency of contact to neighbors (in % of respondents); Source: own survey (*N* = 873–894).

In contrast to the predominantly positive evaluation of the neighborhood quality, contacts among neighbors are of minor intensity (Figure 3). Even though most respondents indicate that they visit their neighbors or lend items to their neighbors, only a minority of respondents states they do this often or very often. However, every second respondent offers help to neighbors either often or very often, which indicates a generally positive and attentive attitude among our respondents. Having a look at the place of living, we observed a slight decrease of the mentioned contact activities with rising settlement size (*r* = −0.09, −0.07, −0.11, *p* = 0.01), which might hint toward more intensive contacts and relationships in small towns.

Refugees rarely mentioned specific expectations of neighborhood relationships. In case they touched upon that issue, they stressed very general expectations, such as safety and security. Moreover, they consider a “good” relationship with their neighbors, which most define as living alongside each other without having major problems, as important. For some, “good” includes having mutual invitations to talk and eat together as was common in their countries of origin. Besides positive expectations, some respondents anticipated possible negative attitudes toward families with noisy children as well as foreigners.

I don't want to cause problems. Anyway, people talk about foreigners. Lots of problems. I actually don't want to cause problems. I simply want to live in peace, making use of this second chance. (male Syrian, in his 20s, II-6^4^)

The refugees' perceptions of neighborhood relationships are negotiated relationally—between their rural site of living and their previous places of residence in other parts of Germany or other countries. In addition, refugees in rural areas perceived differences with regard to neighborhood and daily encounters in urban areas that stem from their own experiences during visits to relatives or on shopping trips. In cities, neighbors are perceived as more anonymous and they and random people on the street do not regularly greet each other, whereas in villages and small towns contacts are more personal:

This is a big advantage in the countryside […] that you can better, I mean more easily get to know people. And you are not a number, but it is personal. (male Syrian, in his 20s, VI-4)

When we go out, other people are always very friendly, for instance our German neighbors. We always greet each other on the streets. (female Afghan, in her 50s, IV-25)

Casual encounters on the streets associated with friendly greetings, such as “Hallo, wie gehts?” (“Hi, how are you?”) are evaluated positively. Some interviewees highlighted the advantage that everyone knows everyone, especially if they need help, while others perceived social proximity to be one-sided and found similarities to rural areas in their home countries:

Here, everyone knows me, but I know nobody [laughs]. This is really … creepy, I don't know [laughs]. This is the same in Syria, if you live in a village. There, everyone knows the stranger, the one who is not from there. (male Syrian, in his 20s, III-3)

In addition, some participants from Saxony even perceived it to be easier to get to know people in cities, as they believed people in their rural places of residence were not so open-minded (e.g., interviews VII-9 and_VIII-5). This may result from a considerably lesser migration-related diversity in the East German case study regions compared to most West German regions (e.g., Bösch and Hong So, 2020).

In other cases, participants perceived that rural inhabitants do not match their age profile. This is an issue if the refugees, who are younger than the average population in rural areas, want to establish contacts with people of the same age. The refugees' reflections and experiences regarding the demographic and ethnic fabric of rural neighborhoods are supported by the survey data, where respondents characterized their neighborhoods as having high proportions of elderly people but also (albeit to a lesser degree) families with children, and as being ethnically homogeneous: over 80% indicate that there are only few inhabitants with a migration biography in their neighborhood (Table 1).

Summing up, residents have a positive impression of their neighborhood and describe neighborhood relations as good, albeit rather functional. Newcomers to the neighborhood are expected to abide by the (unwritten) social rules, e.g., greeting on the streets. This attitude is well-reflected by the refugees, who try to understand how things are done “correctly” and adapt to them (see also findings from Larsen, 2011). Thus, the interviewed refugees display a high level of reflexivity regarding their new neighborhood and how they might be seen by the German residents. They also reflect on the variations of neighborhood relations in Germany regarding age, family status, and ethnicity of their neighbors, and integrate those experiences into an explanatory frame which is strongly shaped by the neighborhood culture in their home countries. Social proximity as a peculiar notion of rural neighborhoods, associated with serendipitous encounters in public space and the absence of anonymity, is confirmed by refugees. Regarding general perceptions about diversity and the level of tolerance in their neighborhood and the municipality as a whole, residents display an assimilative perception of coexistence; this might not (only) represent their personal perception, but the anticipation of the general mood in society, as the differentiated assessment of their municipality's aptness to integrate newcomers with specific profiles (Figure 2) points out.

### Experiences of Daily Encounters and Social Relationships

**Figure 4:**
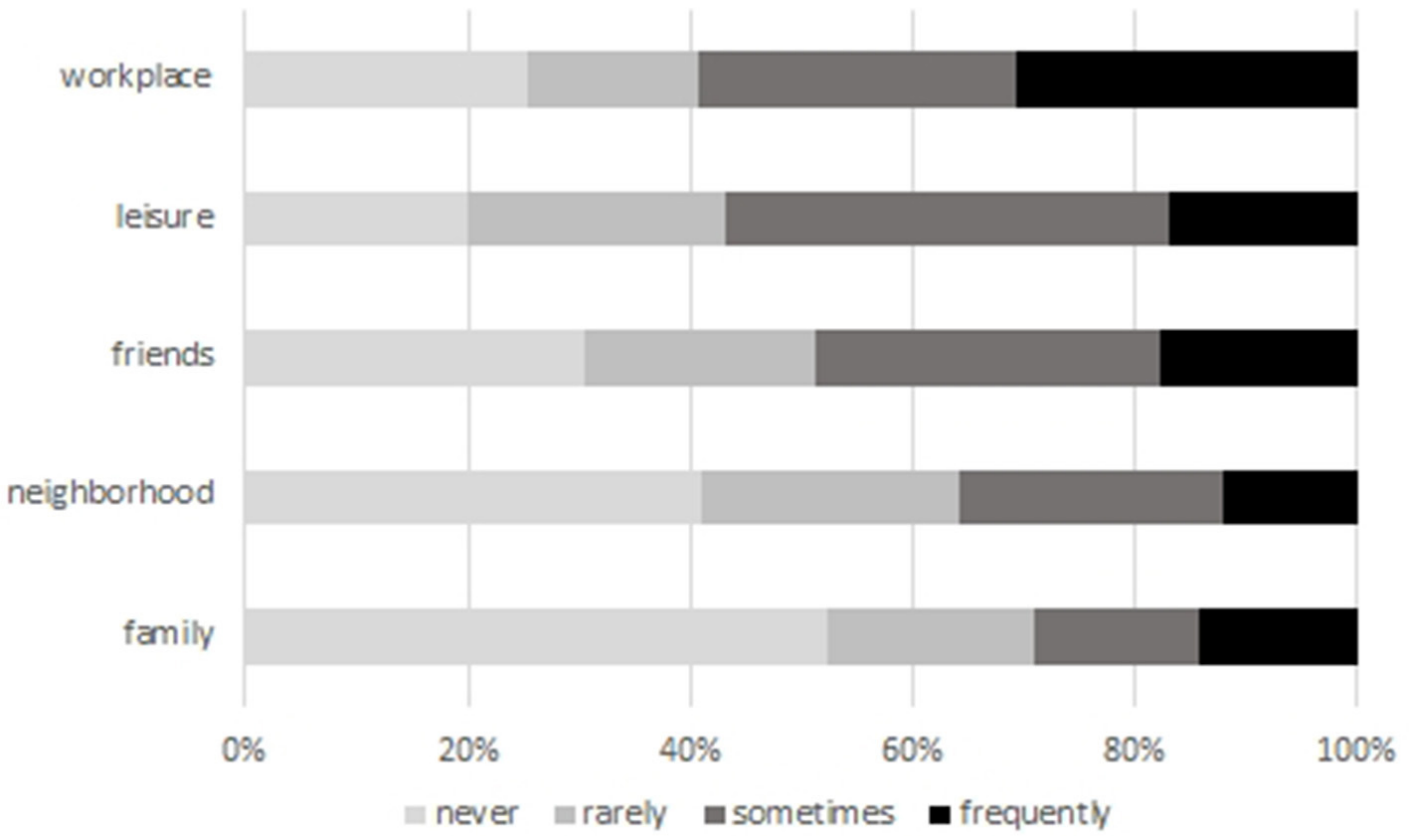
Positive experiences with foreigners in everyday situations (in % of respondents); Source: own survey, *N* = 818–847.

In our next dataset, we asked our respondents about the frequency of their contact with foreigners. Firstly, we wanted to identify weak ties that provide bridges between two or more bounded groups. Secondly, we focused on positive experiences made during such encounters, as various empirical studies showed that especially positively perceived contacts can reduce stereotypes (Pettigrew and Tropp, 2006; Rapp, 2014; Roos, 2016) (Figure 4).

As the data show, positive contact experiences with foreigners are generally rare among our respondents. If there are positive contacts of a significant frequency, these happen mostly in the workplace, or (less frequently) during leisure activities or in circles of friends. On the other hand, a majority of our respondents never or only rarely had any positive experiences with foreigners in their neighborhood or in the family. The prevalence of positive contact stood in close relation to the presence of foreign residents in the respective rural district: while respondents from the two districts with the lowest share of foreigners (below 4%) reported the lowest share of positive contacts (below 1.5%) and the highest share of no positive contact experiences with foreigners in the neighborhood (more than 59%), respondents who reported the highest share of positive contacts (more than 18%) lived in the three districts with the highest share of foreigners (more than 7%). This finding weighs even more when its correlation to xenophobic sentiments is considered: in our sample, those who reported few or no positive contact experiences with immigrants expressed xenophobic sentiments to a larger extent than those with positive contact experiences. Both findings clearly support the contact hypothesis, which assumes that negative stereotypes can be reduced and integration processes be supported when having frequent inter-group contacts in everyday situations (see similar conclusion in Roos, 2016). With regard to the specifics of rural settlements, we can compare our findings with the general population survey ALLBUS, which was collected with a representative sample and thus represents both urban and rural living conditions. The ALLBUS found that 72.4% of all respondents in Germany reported frequent positive contacts with foreigners in various situations, whilst only 10.4% had negative ones. Mostly, contacts occurred in circles of friends (54.2%), in the workplace (51.8%) and in the neighborhood (41.1%, Gesis, 2016). Thus, we need to highlight the absence of a diverse population as an important contextual condition relevant for the well-being of foreign newcomers in rural settings.

While respondents of the citizens' survey had very little direct contact with foreigners at all, the interviewed refugees could certainly all reflect on experiences of social interactions in their direct living environment and beyond. Our interviewees' experiences were rather diverse. While some reported close social contacts and mutual support, others experienced very limited and superficial social contacts, and also negative experiences:

Where we live, we only have little contact with our neighbors. Except for the one neighbor who lives above us, we hardly talk with each other. […] And it not only works like this for us, but we know another family living close. For them it is very similar: they only have little contact with their neighbors. (male stateless person, in his 30s, IV-20)

Explanations for scarce social contacts are firstly a lack of time on the part of both refugees and neighbors due to work, participation in language classes and further education commitments, family obligations or concentrating on cultivating contacts outside the region. Secondly, language barriers may further impede socializing, in case refugees are not yet comfortable with their German language skills or there is no common language in which to talk with other foreigners. Thirdly, there may simply be a lack of possibilities for social interactions, especially if asylum accommodation or private apartments are situated on the periphery of villages or towns, where refugees are the only residents and have no direct neighbors.

Nevertheless, a considerable share of our interviewees reported positive experiences and described their neighbors as “nice” and “friendly”:

And our neighbors, whether Germans or Arabs or whatever, are all nice. Nobody has a problem. (male Syrian, in his 20s, II-6)

We do not have close contact with the neighbors, but in general they are very friendly and the landlord is nice as well. He comes every now and then and checks if everything is fine. (male Syrian, in his 30s, III-31)

While the first quote suggests very limited expectations regarding positive neighborhood relations, i.e., the mere absence of problems, the second example indicates that the typical rural setting of houses with a limited number of tenants or with the landlord living in the same house can be a source of support in everyday matters. This support may encompass occasional looking after kids, assisting with doing homework for school or teaching German language:

The landlord always tried to practice and teach German with our family. And he was always motivated to just talk with us, and read and write. And we—my wife and I, and both our kids—learnt quite a lot from him. We have contact nearly every day, every day we talk with each other. (male Syrian, in his 30s, V-19)

The example shows that social bridges are first and foremost instrumental, confirming the findings of Wessendorf and Phillimore (2019). Moreover, social interaction is supported by spatial proximity, since the landlord is involved. However, in other cases we found that landlords or other tenants were perceived as rather obtrusive with a paternalistic behavior or, conversely, left contact requests unanswered. This points to the ambivalence of the specific fabric of rural neighborhoods regarding the effects of physical nearness and social control.

However, the specifics of rural neighborhoods, notably the expectations regarding the behavior of newcomers to introduce themselves, greet others, and offer help (see Figure 1), can be a starting point for establishing positive relations, if the newcomers are aware of those practices. Our data show a number of positively valued neighborhood relations where refugees are the ones who provide support. Bi-directional contacts where refugees and neighbors are at eye level emerged from casual meetings in the stairway or garden, where kids can act as bridge builders (cf. Stachowski, 2020).

The casual meetings, in turn, pave the way for more intensive situations of social contact, such as inviting neighbors to their own homes.

When I met her first, I said: “Please, have a coffee with me.” She was surprised that I simply said “Please, have a coffee with me.” She said: “The Germans do not do that.” […] But then we drank coffee and ate cake and since then we are very good neighbors. (female Syrian, in her 30s, VII-12)

And I do have here—most important—I have a nice neighbor. I can always have a cup of coffee with her, and a chat with her, and this is my hobby. […] And she takes care of my kids, and I take care of hers. Everything is fine. (female Syrian, in her 20s, I-1)

As the first quote suggests, individual invitations among neighbors may be introduced as a new social practice by the newcomers and can be valued positively by local residents, thus triggering social change in neighborly relations. Also, as the second quote suggests, biographical similarities, such as bringing up children can be an additional incentive for intensified neighborly contact and the development of belonging.

Interviewed refugees also reported motivations for establishing social bridges, e.g., mutual interest in each other's families or customs. As the following quote shows, intercultural openness of the local residents is valued as a sign of welcome by the interviewees.

The inhabitants always celebrate Eid^5^ with us. After the prayers, they came over and we were surprised. In (*place anonymised*) they searched on the internet how Muslims celebrate Eid and then they prepared everything, so that the people did not feel like strangers. (male Syrian, in his 40s, and female Syrian, in her 30s, I-6)

In this case, those initial practices of building social bridges were identified by interviewees, underlining the stages described by the contact hypothesis (social contact-reciprocity-change). Moreover, in this case empathy for biographical crises in the neighborhood seemingly led to the establishment of social bonds, and thus to place attachment:

But we also celebrate festivals with people, and if someone dies, we go to their relatives to comfort them. We do not feel like strangers anymore; we belong to this place now. (male Syrian, in his 40s, and female Syrian, in her 30s, I-6)

Additionally, new institutions established by volunteers also provide important opportunities for social contacts. In particular, refugee relief groups were frequently cited.

Through our German neighbors, through the “Asylcafe,” there is a gathering for women, who always help refugees. Those people had contact with the local administration.

Such institutionalized meeting places provide opportunities for mutual contact on a regular basis and build bridges in both directions. Simultaneously, volunteers attending meetings also operate as actors who connect refugees to state institutions (social links in the sense of Putnam), such as the local administration. Alongside social bridges, social bonds also develop in rural neighborhoods and localities. As the quotes below show, mutual visits were highlighted, whilst small migrant communities were reported, especially in small towns.

We Syrians continuously visit each other, and when you have a problem, you go to your neighbor. (male Syrian, in his 20s, II-2)

A family who came from Turkey chatted a lot with me, provided assistance and visited me. (male Syrian, in his 30s, V-14)

Well I have the advantage that I have many relatives and acquaintances living here. They have been here for years, know how things work here and give me a lot of help. When I need something to do, work, then I find it quickly. (male Syrian, in his 30s, V-10)

Members of the migrant community who have lived in the rural locality for longer play an important role for orientation and knowledge transfer. As such, they take on the role of mediators.

Summing up, the interviewed refugees discuss elaborate experiences regarding bridging social capital, i.e., contacts to local residents in their neighborhood, and among social networks of friends (see also Gilhooly and Lee, 2017). Listening to their accounts, it becomes clear that those kind of social bridges that are evaluated positively are based on mutual interest around family issues or cultural aspects. The latter may be especially applicable for open-minded rural residents, who consider meeting migrants as a chance for intercultural encounters in rural areas that were relatively homogeneous in the past. Simultaneously, social bridging may evoke wider social changes once residents share positive experiences. As rural residents often hold multiple roles in the society, this increases opportunities for encounters. Refugees' narrations also reveal the importance of contacts with their own group in terms of bonding social capital (see also Larsen, 2012; Kilpatrick et al., 2015). While the possibility of finding stability within a community sharing a similar background may be easier in many urban agglomerations compared to rural regions, the effects upon integration are still debated, e.g., in terms of socio-spatial segregation (Daley, 2007; Spicer, 2008; Platts-Fowler and Robinson, 2015).

## Discussion and Conclusion

The scope of this paper was to analyze the role of social contact as a factor in refugees' well-being and place attachment. The assumption was that these might foster positive integration outcomes and long-term settlement, which in turn can contribute to community development. Drawing from an on-going research project on the integration of refugees in rural regions in Germany, we specifically examined expectations, perceptions and experiences of neighborhood relationships and social contact among resident population and refugees. Both datasets illustrate the specific perspectives of rural inhabitants, considering the resident population and refugees at a considerable sample size. The combination of both datasets enables us to indicate social norms, based on expectations, which refugees (have to) adapt to, as well as neighborhood practices and experiences. The latter were mostly reflected upon by refugees; they could identify social contacts that contribute to well-being. We will now wrap up the main findings of our empirical study and reflect them against the backdrop of our guiding questions and concepts.

Regarding the openness of the resident population toward foreign newcomers and the level of tolerance, we used the contact hypothesis to create our guiding questions. We assumed that the possibility to meet foreigners on an everyday basis would help to reduce stereotypes, while the absence of those contact opportunities might hamper the development of a tolerant position. We showed that among our rural survey respondents, there are only few experiences of everyday contact with foreigners, not only in their neighborhood, but in general. This is undoubtedly due to the low level of diversity in most parts of our surveyed areas, which lowers the probability for intercultural encounter. Moreover, we found correlations between the scarcity of inter-group contacts and the expression of negative stereotypes against immigrants. Those findings strongly support the contact hypothesis. With regards to our guiding assumptions on the assets of rural reception conditions, we could identify an ambivalence regarding social proximity and control, and the necessity to consider not only the quantity but also the quality of social contacts.

Expectations of a good neighborhood quality encompass calm and helpful attitudes and, especially among resident populations, culturally mediated expectations, such as greeting on the street, and activities which newcomers are expected to adjust to. Regarding perceptions of rural neighborhoods, refugees confirmed that there is social proximity, especially in relation to urban areas. Social bridges often include weak ties and serendipitous encounters. Whilst Wessendorf and Phillimore (2019) suggest the functional character of these sorts of interactions, and assume that primarily closer friendships evoke a sense of belonging, our data suggest differentiating this in the specific context of the neighborhood. First, the intensity of social interactions are dynamic since casual encounters with neighbors may become intensified and result in acquaintances and friendships. Weak ties may become strong ties depending on (1) time spent together, (2) degree of emotional intensity, (3) intimacy (mutual trust), and (4) the type of reciprocal assistance (Granovetter, 1973). Second, individuals can ascribe meaning to casual encounters and consider them as a contribution to attachment (cf. Ager and Strang, 2008). Such weak ties to native residents, but also to other foreigners and refugees who arrived in the past, are evaluated as satisfying in cases where refugees had heard about or initially feared bad interactions with neighbors. They are considered important when it comes to overcoming “everyday otherness” and long-term assistance with regard to the integration process (see also structural and transversal enablers, Radford, 2016), as well as for the development of a group identity. The question of whether strong ties represent a precondition for long-term settlement cannot be affirmed yet. Regardless, individual aspirations and the opportunities for social interactions as well as the agency to create opportunities are crucial. Investing in a good neighborhood as one part of well-being may be considered as a first and important step toward the development of staying aspirations. For this purpose, a continuous reflection on aspirations and what the current place of residence can provide (place dependence), as well as emotional attachment, is necessary.

## Data Availability Statement

The datasets presented in this article are not readily available because datasets are confidential as agreed upon with the study participants. However, they can be made available on site upon request. Requests to access the datasets should be directed to johanna.fick@thuenen.de.

## Ethics Statement

The studies involving human participants were reviewed and approved by Ethikkommission der Philosophischen Fakultät der TU Chemnitz. The patients/participants provided their written informed consent to participate in this study.

## Author Contributions

BG was involved in drafting the paper idea and structure of the article and writing of the introduction, discussion and conclusion jointly with co-authors, wrote the conceptual considerations (especially chapter Social Contact and the Role of the Resident Population), the method section (chapter Representative Survey) as well as the results section on the survey data, and made substantial contributions to the revision procedure and was in charge for communication with the reviewers and editors throughout the submission and review process. SK was involved in drafting the paper idea and structure of the article and writing of the introduction jointly with co-authors, drafted and wrote conceptual considerations (especially The Role of the ‘Social’ Regarding Refugees' Well-being and Place Attachment) and the results section regarding qualitative data from refugees, and also drafted and wrote discussions and conclusions jointly with co-authors and made substantial contributions to the revision process. TW drafted the paper idea jointly with co-authors and contributed substantially to the writing of material and methods section (Interviews With Refugees) as well as the elaboration of the results section with regard to the refugee perspective and involved in the revision process and made substantial contributions. MB, HS, and DS were involved in the analysis of data and drafting the structure of the results section and made substantial contributions throughout the whole revision process. All authors contributed to the article and approved the submitted version.

## Conflict of Interest

The authors declare that the research was conducted in the absence of any commercial or financial relationships that could be construed as a potential conflict of interest.

## Footnotes

^1^The collaborative research project “Future for refugees in rural regions of Germany” is a collaboration between the Chemnitz University of Technology, the University of Erlangen-Nuremberg, the University of Hildesheim and the Thuenen Institute of Rural Studies.

^2^The indicator considers population density, share of agriculture and forestry, share of one- and two-family houses, inhabitants in the catchment area and distance to major urban centers (Küpper, 2016, p. 5).

^3^The sample mainly consisted of individuals who had received a temporary or permanent protection status due to their asylum application (entitlement to asylum, refugee protection or subsidiary protection, or a national ban on deportation), or for other reasons, e.g., due to resettlement, as well as 25 individuals who had had a suspension of deportation following a rejection of their asylum claim, or permission to reside as they were still in their asylum procedure.

^4^To protect personal data of our participants, only vital data are displayed here. For reasons of transparency, however, all quotes presented are tagged with a token including the rural district and the interview number.

^5^Eid-al-Fitr is a religious holiday that marks the end of the fasting month Ramadan.
